# Impact of blending the genomic relationship matrix with different levels of pedigree relationships or the identity matrix on genetic evaluations

**DOI:** 10.3168/jdsc.2022-0229

**Published:** 2022-07-26

**Authors:** Mary Kate Hollifield, Matias Bermann, Daniela Lourenco, Ignacy Misztal

**Affiliations:** Department of Animal and Dairy Science, University of Georgia, Athens 30602

## Abstract

Evaluations using single-step genomic BLUP require blending the genomic relationship matrix (**G**) with a positive definite matrix to ensure nonsingularity for solving the mixed model equations. Many organizations blend **G** with a proportion of the numerator relationship matrix for genotyped animals (**A**_22_) to improve stability and possibly add a residual polygenic effect. However, when nearly all the polygenic variance is explained by **G**, blending with **A**_22_ may cause inflation and add excess computing time; thus, blending with an identity matrix (**I**) multiplied by a small value may be a better solution. The objective of this study was to evaluate changes in reliability and inflation of genomic estimated breeding values, convergence rate, elapsed wall-clock time for blending **G** with different levels of **A**_22_ or **I**, and develop a more time-efficient blending method. A US Holstein cattle data set was used with 9.7 million animals in the pedigree, 569,404 animals with genotypes, and 10.1 million stature phenotypes. Blending **G** by adding a small value to the diagonal elements had comparable performance to **A**_22_ with fewer rounds to convergence required to solve the system of equations. Reliability and inflation of genomic estimated breeding values ranged from 0.63 to 0.68 and 0.86 to 0.89 for all blending scenarios tested. The current blending default in the BLUPF90 software is to replace **G** with (1 – *β*)**G** + β**A**_22_, where *β* equals 0.05. In this study, *β* values of 0.30, 0.20, 0.05, 0.01, 0.005, and 0.001 were evaluated with **A**_22_ and **I**. Negligible differences in elapsed computing time between the blending types and levels were observed. Subsequently, the current blending algorithm used in the BLUPF90 family of programs was optimized, reducing the blending time from approximately 2 h to 5 min for **A**_22_ and less than 1 s for **I**. The new time difference between blending with **A**_22_ or **I** is negligible and not computationally critical. The results indicate that blending **G** with **A**_22_ does not have clear advantages over blending with a small proportion of **I**.

## Highlights

• Blending **G** with **I** had negligible differences in reliability and inflation of GEBV compared with **A_22_**.
• Fewer rounds to convergence were required when **G** is blended with **I** compared with **A_22_**.
• The optimized blending algorithm is several orders of magnitude faster than the old algorithm.
• The elapsed time for blending is not computationally critical with the optimized algorithm.

## Graphical Abstract

**Figure:**
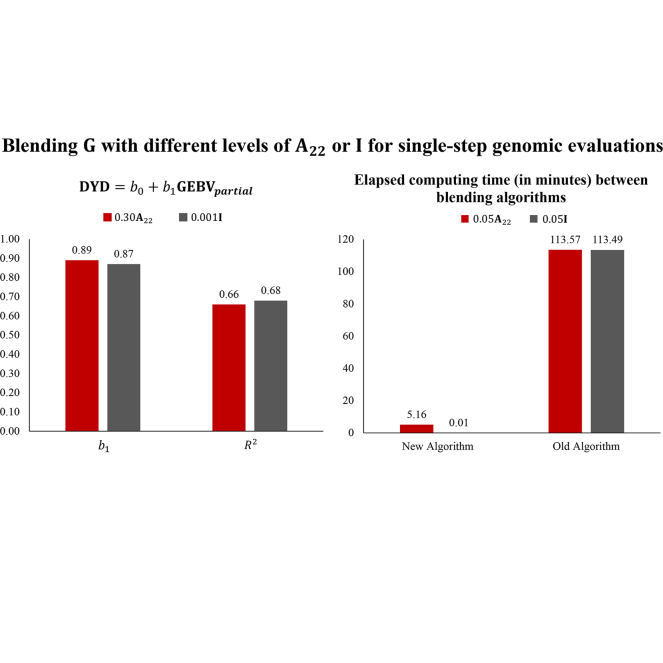
**Summary:** For single-step genomic BLUP, the genomic relationship matrix (**G**) is blended with a positive definite matrix for inversion to solve the mixed model equations. Conventionally, **G** is blended with a proportion of the numerator relationship matrix for genotyped animals (**A_22_**); however, blending with **A_22_** can take excess time, whereas blending with an identity matrix (**I**) may take less time and guarantees the nonsingularity of **G**. The purpose of this study was to compare differences in the reliability and inflation of genomic estimated breeding values (GEBV), convergence rate, and elapsed wall-clock time when blending **G** with different proportions of **A_22_** or **I** and introduce a more efficient blending algorithm. Using a US Holstein population of 9.7 million animals in the pedigree and 569,404 genotypes, negligible differences in performance were observed in blending with 0.001I and 0.30**A_22_**. The optimized blending algorithm reduced the computing time from approximately 2 h to 5 min for **A_22_** and less than one second for **I**.

Single-step genomic best linear unbiased predictor (**ssGBLUP**) allows estimating the breeding values jointly for genotyped and nongenotyped animals (Aguilar et al., 2010; Christensen and Lund, 2010). For solving the mixed model equations (**MME**), the main difference between ssGBLUP and the pedigree-based best linear unbiased predictor (**PBLUP**) is in the covariance matrix for the breeding values. In PBLUP, the inverse of the numerator relationship matrix (**A**^−1^) is used, whereas in ssGBLUP is replaced by **H**^−1^. The matrix **H**^−1^ is composed of **A**^−1^, the inverse of the genomic relationship matrix (**G**^−1^), and the inverse of the numerator relationship matrix for genotyped animals $\left(A_{22}^{-1}\right)$ as follows:

[1] $$H^{-1}=A^{-1}+\left[\begin{array}{cc} 0 & 0\\ 0 & G^{-1}-A_{22}^{-1} \end{array}\right].$$

Calculating such matrices can be referred to as the “genomic setup,” which typically involves computing **G**, adding (“blending”) it to a small fraction of a positive definite matrix; usually an identity matrix (**I**) or **A**_22_, to guarantee its nonsingularity (VanRaden, 2008), tuning to make it compatible with **A**_22_ (Vitezica et al., 2011), and inverting it. The current default in BLUPF90 is blending implemented first and then tuning. McWhorter et al. (2021) found predictions were unbiased, accurate, and neither over- nor under-dispersed with either order. When solving the MME, **A**^−1^ is calculated following Henderson's rules (Henderson, 1976; Quaas, 1976, 1988). For small evaluations, **A**_22_ is calculated with Colleau (2002) and inverted, whereas for large-scale evaluations, a product $(A_{22}^{-1})\text{q}$ is calculated as proposed by Masuda et al. (2016). Typically, **G** is constructed using the first method of VanRaden (2008):

[2] $$G=\frac{ZZ\prime }{2\sum p_{j}\left(1-p_{j}\right)},$$

where **Z** = **M** − **P**, *p_j_* is the allele frequency of the second allele at locus *j*, calculated based on observed allele frequencies, **M** is a genotypic matrix relating marker alleles to individuals with the number of rows equal to the number of animals and the number of columns equal to the number of SNP, and **P** is a matrix containing 2*p_j_*.

The blending step is frequently done as (1 − *β*)**G** + *β***A**_22_, where *β* reflects the proportion of residual polygenic variance not accounted for by **G** (Habier et al., 2007; Liu et al., 2016; Mäntysaari et al., 2017). Blending in this way creates a nonsingular **G** and is equivalent to fitting a residual polygenic effect (**RPG**) in the model (Liu et al., 2014). However, when nearly all QTL were identified, using **A**_22_ for blending reduced accuracy while using a fraction of **I** for blending did not (Fragomeni et al., 2017). The results from the study of Himmelbauer et al. (2021) showed that blending with *β* greater than 0.001 introduced biases for bulls with many genotyped progeny. It is standard for *β* to be equal to 0.05; although, depending on the population parameters and quality of genomic information, values of *β* can vary from 0.01 to 0.50 (Lourenco et al., 2020). Larger values of *β* are used when the markers do not adequately explain the additive genetic variance or to reduce the effect of the genomic information (Meyer et al., 2018).

When the number of genotyped animals is large, **G**^−1^ cannot be computed. In such a case, **G**^−1^ can be replaced by $G_{APY}^{-1},$ which is calculated with the algorithm of proven and young (**APY**; Misztal et al., 2014; Misztal, 2016). Let **G** be partitioned as

[3] $$G=\left[\begin{array}{cc} G_{cc} & G_{cn}\\ G_{nc} & G_{nn} \end{array}\right],$$

where the subscripts *c* and *n* denote the blocks for core (proven) and noncore (young) animals, respectively. Using APY, the inverse of $G\left(G_{APY}^{-1}\right)$ can be obtained directly as

[4] $$G_{APY}^{-1}=\left[\begin{array}{cc} G_{cc}^{-1} & 0\\ 0 & 0 \end{array}\right]+\left[\begin{array}{c} -G_{cc}^{-1}G_{cn}\\ I \end{array}\right]M_{nn}^{-1}\left[\begin{array}{cc} -G_{nc}G_{cc}^{-1} & I \end{array}\right],$$

where $M_{nn}=diag\left(G_{nn}-G_{nc}G_{cc}^{-1}G_{cn}\right)$ is a diagonal matrix.

The APY reduces computational costs by utilizing concepts of effective population size and the limited dimensionality of the genomic relationship matrix (Fragomeni et al., 2015; Masuda et al., 2016; Pocrnic et al., 2016). Previous studies have successfully tested APY in various species (Ostersen et al., 2016; Mäntysaari et al., 2017; Gonzalez-Peña et al., 2019; Nilforooshan and Lee, 2019). For Holsteins, 15,000 eigenvalues corresponded to 98% of the variation and realized accuracies peaked using this number of randomly chosen core animals (Pocrnic et al., 2016). In addition to APY, other options are available for single-step with large genomic data, such as ssGTBLUP (Mäntysaari et al., 2017), single-step Bayesian regression (Fernando et al., 2014), single-step SNP-BLUP (Liu et al., 2014; Taskinen et al., 2017), and using reduced-dimension singular value decomposition of the genotype matrix (Ødegård et al., 2018). Although APY makes it possible to obtain a sparse representation of **G**^−1^ $\left(G_{APY}^{-1}\right)$ that well depicts the population structure without inverting the entire **G** directly, blending is still needed because of the inverse of **G** for core animals. The computing time of the genomic setup may be a limiting factor to timely accomplish large-scale evaluations when the number of genotyped animals exceeds 1 million. Therefore, this study aimed to compare the efficiency of blending **G** with **I** versus **A**_22_, analyze the reliability and inflation of the resulting genomic estimated breeding values (**GEBV**), and develop an improved blending algorithm.

The current implementation of APY in preGSf90 follows the methods in Masuda et al. (2016), and at the time of the development, the number of genotyped animals was small; therefore, the algorithm was efficient. Now that the number of genotyped US Holsteins is nearing 5 million (Council of Dairy Cattle Breeding, 2022), a more efficient blending method is required for feasible routine evaluations. Because of the structure of $\left(G_{APY}^{-1}\right)$ and for memory efficiency, only **G***_cc_* and **G***_cn_* are stored as dense matrices, whereas for **G***_nn_*, only its diagonals (**g***_n_*) are stored. In the blending proposed by Masuda et al. (2016), all the columns of **A**_22_ are calculated, but only the elements corresponding to **G***_cc_*, **G***_cn_*, and **g***_n_* are added to these matrices. The rest of the elements are used for calculating the average of the diagonal and off-diagonal elements of **A**_22_, which are required for the tuning of **G**. Since the only elements of **A**_22_ needed are those corresponding to **G***_cc_*, **G***_cn_*, and **g***_n_*, we propose an optimized algorithm that computes only the rows of **A**_22_ corresponding to the core animals instead of calculating all the columns of **A**_22_. The elements of **A**_22_ corresponding to **g***_n_* are the inbreeding coefficients added to the value of one for the noncore animals. They are calculated before computing any row of **A**_22_ because the method for calculating these rows requires the inbreeding coefficients (Colleau, 2002).

The primary purpose of blending **G** with **A**_22_ is to make **G** nonsingular, which is attainable with **I**. Blending with **A**_22_ may cause unwanted bias, and blending with **I** should require less computing time. To evaluate this, we compared the reliability and inflation of GEBV, number of rounds required for convergence, and elapsed wall-clock time for blending when blending **G** with various proportions (0.30, 0.20, 0.05, 0.01, 0.005, 0.001) of **A**_22_ and **I**. A US Holstein data set provided by The Council on Dairy Cattle Breeding (Bowie, MD) was used in this study. Stature phenotypes were available from 10,067,745 animals. The pedigree file included 9,730,943 animals, from which 569,404 animals had 60,671 SNP markers after quality control. Single nucleotide polymorphisms with minor allele frequency lower than 0.05, call rates lower than 0.9, or a difference greater than 0.15 between expected and observed frequency of heterozygous were removed during quality control. Animals with call rates lower than 0.9 or parent-progeny Mendelian conflicts were removed during quality control. Of the genotyped animals included after quality control, 21,127 were sires, 59,723 were dams, and 32,855 had stature phenotypes. For APY, 15,000 genotyped animals were randomly chosen as core.

A partial data set was created for validation by removing phenotypes from daughters of bulls that have at least 50 daughters with records in 2014. Genomic EBV (GEBV) were calculated for the whole (GEBV_w_) and partial (GEBV_p_) data sets using the BLUP90IOD2OMP1 software (version 3.119; Tsuruta et al., 2001; Tsuruta and Misztal, 2008). Estimates of daughter yield deviations (**DYD**) for validation bulls were obtained using the whole data set with the method of Liu et al. (2004) and the algorithm of Mrode and Swanson (2004). The regression coefficient (*b*_1_) and the coefficient of determination (R^2^) between DYD and GEBV_p__x were used to measure the inflation and reliability of predictions for validation bulls, respectively, where x denotes the blending combination tested (0.30, 0.20, 0.05, 0.01, 0.005, or 0.001 multiplied by **A**_22_ or **I**).

The results for the validation are shown in Figure 1. None of the *b*_1_ nor R^2^ values between DYD and GEBV_p__x differ by more than 0.05, indicating consistent outputs of the models. The lowest R^2^ values were seen with the blending coefficient of 0.30 for both matrices tested. To compare the GEBV of the various blending combinations to the current blending default in the BLUPF90 programs (0.05**A**_22_), GEBV_w__0.05**A**_22_ was regressed on GEBV_w__x, and the correlation coefficients (*r*) and *b*_1_ of the 2 were calculated for the genotyped animals and are shown in Figure 2. When comparing with the same blending proportion, there were negligible differences between *b*_1_ and *r* for **I** and **A**_22_. For GEBV_w__0.05**A**_22_ regressed on GEBV_w_ of 0.30, 0.20, 0.01, 0.005, or 0.001 **A**_22_ blending combination, *r* and *b*_1_ ranged from 0.99 to 0.98 and 0.99 to 0.97, respectively, indicating very little inflation and strong association. For GEBV_w__0.05**A**_22_ regressed on GEBV_w_ of each **I** blending combination, *r* was 0.99 and *b*_1_ ranged from 0.98 to 0.97. The differences observed here are negligible and suggest no differences in reliability or inflation of GEBV when blending **G** with a small value multiplied by **I** compared with **A**_22_.

**Figure 1:**
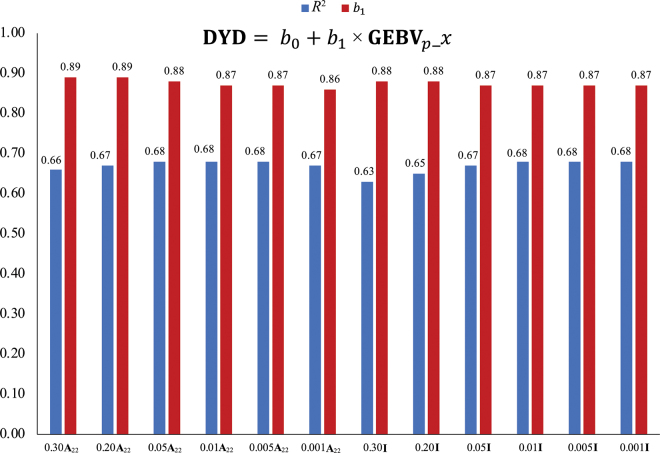
Regression coefficient (*b*_1_) and coefficient of determination (R^2^) of daughter yield deviations (DYD) on genomic EBV calculated with a partial data set (GEBV*_p_*) with all GEBV*_p_*_*x* blending scenarios, where *x* denotes the blending combination tested, as shown on the x-axis. **A_22_** = numerator relationship matrix for genotyped animals; **I** = identity matrix; *b*_0_ = intercept of the regression line.

**Figure 2:**
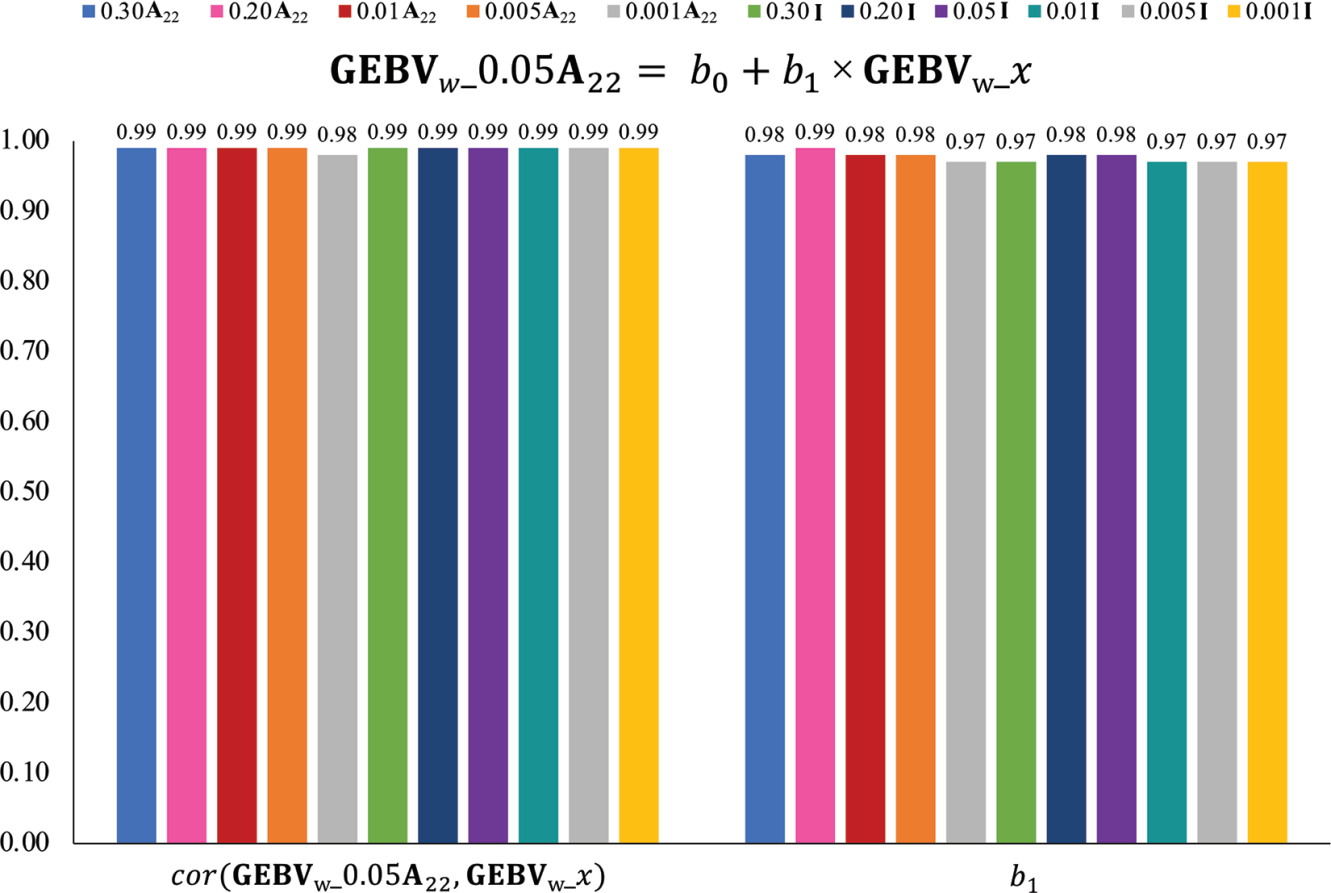
Regression coefficient (*b*_1_) and correlation coefficient (*r*) of GEBV*_w_* using 0.05**A**_22_ blending on GEBV*_w_*_*x*, where *x* is the blending combination tested as shown in the legend and *w* refers to the whole data set. GEBV = genomic estimated breeding values; *b*_0_ = intercept of the regression line.

The number of rounds until convergence using preconditioner conjugate gradient with iteration on data (Tsuruta et al., 2001) were compared for each blending combination to quantify the computational efforts and are shown in Table 1. The termination criterion was 10^−12^ with a convergence criterion of C = **b** − **Ax**^2^/**b**^2^, where the MME are **Ax** = **b**. For every blending combination, the convergence patterns were steady, and there was no indication of divergence. The 0.001**A**_22_ and 0.001**I** blending combinations had the most iterative rounds to convergence (599 and 596, respectively). The blending scenario of 0.30**I** took 251 rounds to converge, which was the fewest observed (Table 1). The fewer rounds necessary for convergence suggest a more well-conditioned system of equations. However, the results do not indicate a concerningly high number of rounds or a diverging system for any blending combinations tested.

**Table 1:** The elapsed wall-clock time for blending in minutes and the number of rounds to reach the convergence criterion of 10^−12^ for obtaining the solutions of the system of equations for each blending scenario with the new and old algorithm^1^

Blending scenario	Algorithm	Elapsed wall-clock time for blending (min)	Rounds to convergence^2^
0.30**A**_22_^3^	New	2.4	333
	Old	54.6	333
0.20**A**_22_	New	2.9	349
	Old	66.0	346
0.05**A**_22_	New	5.2	416
	Old	113.6	418
0.01**A**_22_	New	2.5	487
	Old	61.0	487
0.005**A**_22_	New	2.3	536
	Old	98.7	534
0.001**A**_22_	New	3.9	599
	Old	98.0	594
0.30**I**^4^	New	2.6	251
	Old	62.2	251
0.20**I**	New	3.0	257
	Old	61.9	257
0.05**I**	New	<0.1	362
	Old	113.5	362
0.01**I**	New	<0.1	470
	Old	111.3	468
0.005**I**	New	<0.1	530
	Old	77.2	532
0.001**I**	New	<0.1	596
	Old	76.0	598

1 The blending scenarios are the matrices added to (1 – β)**G** to obtain an invertible **G**, where **G** = genomic relationship matrix, and β = the proportion of residual polygenic variance not accounted for by **G**.

2 The average elapsed wall-clock time per iteration round was 7.3 s for all scenarios.

3 Numerator relationship matrix for genotyped animals.

4 Identity matrix.

One would assume blending with **I** would drastically reduce computing time since **I** is sparse, and the direct creation of **A**_22_ would be avoided. Using the algorithm by Masuda et al. (2016), the elapsed wall-clock time for creating and blending 0.05**A**_22_ and 0.05**I** with 0.95**G** were both around 1 h and 54 min, with no notable difference (Table 1). This lack of difference in computing time between blending with the 2 matrices can be attributed to the algorithm in Masuda et al. (2016); as mentioned above, all columns of **A**_22_ were calculated for the tuning of **G**, regardless of the matrix used for blending. In contrast, only the rows of **A**_22_ relating to core animals were calculated in the optimized algorithm, which reduced computing time. With the optimized blending algorithm, blending 0.95**G** with 0.05**A**_22_ took 5 min, and with 0.05**I**, it took less than 1 s. Blending **G** for large-scale evaluations is efficient with the optimized algorithm using either **A**_22_ or **I**. Although blending with **I** is remarkably faster than with **A**_22_ using the new algorithm, an elapsed computing time of approximately 5 min is not critical. Additionally, the peak memory use was 78.57 GB and did not differ between models.

The new algorithm can include virtually any number of genotyped animals in the genomic setup for ssGBLUP with APY. Using 0.001**I** for blending is enough for inverting and has no negative consequences on reliability and inflation. Moreover, the weight applied to the blending matrix should be determined by the portion of the genetic variance the markers explain. The results may differ depending on the data set and values of *β* used. For each blending proportion tested, **I** had fewer convergence rounds than **A**_22_, and the least was with 0.30**I**. The differences in reliability and inflation of GEBV when blending **G** with various proportions of **A**_22_ and **I** were negligible, and the computing time is no longer a limiting factor with the new algorithm. Therefore, the decision of which matrix to use to ensure the nonsingularity of **G** is trivial for the implementation of ssGBLUP.
